# ‘I think youtube’s turning me into a flat earther’: Social media’s role in ex-conspiracy theorists entering and exiting anti-scientific communities

**DOI:** 10.1371/journal.pone.0323436

**Published:** 2025-06-16

**Authors:** Chanais Matthias, Yael Benn, Ben Harkin

**Affiliations:** Department of Psychology, Manchester Metropolitan University, Manchester, United Kingdom; University of Maryland at College Park, UNITED STATES OF AMERICA

## Abstract

Conspiracy beliefs erode trust in science and lead to negative effects on public health and other science-related behaviours and political discourse. Despite extensive research on conspiratorial thinking, the literature lacks a comprehensive exploration of individuals’ experiences as they enter (and exit) conspiracy communities, presenting a notable research gap. Therefore, the present study adopts an interpretivist framework by interviewing four ex-conspiracy theorists, delving into what drew them into conspiratorial thinking, the catalysts for their exit from these online communities (e.g., Flat Earth, distrust of science and medicine), and the obstacles they faced in disengaging from these communities. Reflective Thematic Analysis identified four main themes with eight associated subthemes. The experiences of ex-conspiracy theorists revealed a profound personal dimension for them entering the conspiracy echo chamber (Theme 1), such as feelings of loneliness and the impact of significant life events. Scientific illiteracy and a general misunderstanding of the scientific process (Theme 2), further contributed to their adoption of more entrenched conspiratorial thinking. The (online) conspiracy community, with its powerful and dynamic nature, had impacted the experiences of members (Theme 3), providing a sense of identity, reinforcing community doctrine, and creating a social and personal barrier to leaving the community. Leaving the community proved a challenging process (Theme 4), with participants identifying a conflict between their personal experiences and the community’s doctrine as a key reason for their departure. We contextualize these findings within prior research and propose potential interventions for individuals susceptible to scientific misinformation, utilizing the principles of nudge theory for behaviour change and mindfulness-based therapies.

## Introduction

### Trust in science and misinformation on youtube

Trust in science (TIS) is fundamental to a healthy democratic society, and when devalued, it threatens public welfare and erodes institutional integrity [1]. TIS refers to the extent to which non-experts value and accept scientific findings, shaping how individuals perceive and engage with scientific information [2]. For example, from 2020 to 2023, amid the COVID pandemic, a 15% decline in trust in science was reported, with 27% of people expressing little to no confidence in scientists to act in the public’s best interests [3]. This erosion of trust has significant implications for public health, particularly regarding vaccination acceptance.

Misinformation, driven in large part by online conspiracy communities, plays a significant role in this erosion. YouTube has become a hub for spreading false claims, such as HIV denial in South Africa [4,5] and the promotion of bleach as a “cure” for autism in the United States [6–8]. This misinformation, coupled with low TIS, is strongly linked to decreased vaccination acceptance, one of the most visible outcomes of scientific distrust. For example, measles outbreaks surged in 17 countries across the WHO European Region in early 2023, exceeding the total number of cases reported in 2022, in part due to the false claim linking the MMR vaccine to autism [9,10]. Despite the WHO debunking this claim in 2003, surveys revealed that 14% of parents in Ontario, Canada, still believed in the vaccine-autism link, with another 14% undecided [11]. This mistrust contributed to a 20% increase in global measles-related deaths by 2017, equating to 110,000 fatalities [12]. Importantly, Mubarak et al. [13] observed, vaccine hesitancy, fuelled by conspiracy theories and the widespread dissemination of misinformation through social media platforms like YouTube, Facebook, X, and Instagram, had devastating public health consequences for large numbers of the Pakistani populace. Li et al. [14] reported ~11% of YouTube’s most-watched videos on COVID-19 vaccines, amassing 18 million views, contained information that contradicted guidance from the WHO and the Centers for Disease Control and Prevention. In addition, Humprecht and Kessler [15] highlighted in their content analysis of 450 YouTube videos on COVID-19 vaccination that the platform contains significant misinformation, including conspiracy theories. The stated specifically that they “predominantly identified completely false YouTube videos about COVID-19 vaccination, characterized by conspiracy theories, anti-elitism, and misinformation about vaccine side effects and safety. These videos aim to create doubt and mistrust by suggesting malicious motives behind the vaccine development and accusing news media of complicity” (p.7).

Given the identified influence of social media platforms such as YouTube on public health behaviours and attitudes toward scientific information through conspiracy theories, it is crucial to address misinformation and its attraction within anti-scientific communities. To this end, the present study fills a gap in the literature by examining the experiences of former conspiracy theorists, exploring key experiences underlying their engagement and disengagement with conspiracies via YouTube. By identifying key influences on their involvement and exit from these communities, we offer insights into the interplay between deeply personal and emotional experiences of participants, trust in science, misinformation, and coercive groups dynamics on social media platforms such as YouTube.. These findings can help inform preventative strategies aimed at rebuilding public trust in science and encouraging healthier behaviours.

### Drivers of engagement and disengagement from conspiracy communities

Expanding on the role of scientific literacy and its influence on public behaviour, extensive research has delved into the dynamics of misinformation. For example, the illusory truth effect refers to the observation that simple repetition of, or exposure to any information, including false or misleading information, increases the likelihood that a person will perceive it as factual, irrespective of its actual truth [16]. A phenomenon shown to be consistent for both oral [17] and written [18] information and operates independently of the credibility of a given source [19,20]. Alarmingly, the illusory truth effect is robust enough to override known truths and proves resistant to correction [21].

Michie et al. [22] suggested that changes in behaviour are more likely to occur when shared verbal or visual information is perceived to be coming from a credible source. This principle was widely used during COVID-19, when governments globally utilized scientific professionals to deliver critical health information through regular public briefings. Despite Abraham and Michie [23] providing a clear definition of a ‘credible source’ (i.e., government entities, health care professionals such doctors, or experts in a field), the perception of the source’s credibility and value may differ between individuals [24]. Moreover, certain untrustworthy sources attempt to create an illusion of trustworthiness by imitating legitimacy. This may involve individuals wearing lab coats to give the impression of being scientists or medical professionals and replicating the format of credible sources like journal articles to enhance perceived reliability [25].

In recent years, social media platforms such as YouTube have become increasingly popular for sharing scientific information. For example, within the UK, YouTube and Facebook stand out as the predominant social media and video platforms, with 91% of internet users aged 15 years and above using them [26]. Since many social media platforms, including YouTube, are not subject to editorial control, they rely on the expertise and intent of the content publisher to provide accurate scientific information [27]. Findings reveal that independent content providers upload ~94% of misleading videos on YouTube [28–30]. This presents a challenge, as social media channels provide a platform for sharing, repeating, and endorsing misinformation, with research identifying YouTube as one of the driving sources of scientific misinformation [28,29,31].

Another reason for the suitability of social media in spreading misinformation is the observation that false or fake news spreads faster than truthful information. Vosoughi, Roy and Aral [32] conducted a large-scale systematic analysis of ~126K tweets from ~3 million people and compared the spread of false versus true news stories (e.g., politics, urban legends, science, and technology). False statements were re-posted quicker, reached more people, were emotionally more evocative, and had more viral qualities than true news stories. Contrary to a popular misconception, people drove the spread of misinformation on social media rather than underlying algorithms favouring incorrect over correct information. Similarly, Bora et al. [28] reported that viewers of videos on YouTube were more likely to share and like videos with false information than videos with accurate information. Therefore, it is evident that disinformation has a unique appeal to those who hear it, with many keen to quickly share it with others [16].

Worryingly, false scientific and medical information often remains uncorrected and accessible for prolonged periods on social media and YouTube [33]. For example, research on medical information on YouTube found that 25% of videos on COVID-19 [34,35], 30% of videos on anorexia [i.e., promoting pro-anorexia; 36] and 38% of those on asthma [e.g., eating live fish as a treatment; [37] provided misleading or incorrect information. Tingley and Wagner [38] reported further examples of conflict between experts and non-experts concerning scientific information, wherein 99% of atmospheric and geochemist experts found no support for a large-scale covert governmental “chemtrail” program, whereas ~60% of the discussions on social media (e.g., YouTube, Twitter) supported this conspiracy theory. In relation to the COVID-19 pandemic, ~ 60% of those who broke the rules and went outside with COVID-19 symptoms, or believed the virus was due to 5G radiation, based their decision on information from YouTube [39].

The proliferation of misleading scientific information on social media creates a considerable barrier to those who wish to engage the public on scientific matters [40]. In this environment, those with limited or no background in science or scientific methodology can quickly find and spread misinformation and distrust in science, technology and medicine [41]. Consequently, social media platforms can result in communities of non-experts sharing information with others of limited scientific backgrounds, which results in echo chambers that reinforce the validity of non-scientific information [42]. Furthermore, the creation of echo chambers on social media not only amplifies the spread of misinformation but also fosters an environment conducive to conspirational and anti-scientific thinking. For example, Diaz Ruiz and Nilsson [43] noted that on social media, a key method was that of ‘echoing’, which enlists participants to spread misinformation through pre-existing controversies and identity-driven culture wars. These findings identify that a lack of (or disregard for) knowledge and rigorous scientific methodologies is associated with the spread of conspiratorial and anti-scientific thinking [44].

### The complexity of motivations behind conspiracy theory engagement

Despite this, mere exposure to, and sharing of, misinformation are not prerequisites to following conspiracy theories or joining their communities. Instead, a bias for conspiratorial thinking arises from the combined influence of various factors, such as mistrust or paranoia toward government services and institutions, cynicism, feelings of political powerlessness, and general defiance of authority [45]. However, these are not solely responsible for people adopting a given conspiracy theory, with numerous additional factors identified in the literature [46]. For example, in the US, political affiliation and race [47,48], or religious views in Poland [49] and Australia [50], have been reported to influence the tendency towards conspiracy-theory based beliefs. In contrast, ‘Science intelligence’, defined as a combination of analytic thinking, quantitative reasoning, and knowledge of scientific facts, was shown to be protective against the influence of non-scientific (flat earth) videos on YouTube, even among those with a strong conspiracy bias [46].

Still, what draws people into, and keeps them immersed in conspiracy theories on social media platforms such as YouTube is not fully elucidated in the literature. Furthermore, there is little understanding of what may help individuals abandon their conspiracy beliefs and what can help them rehabilitate into mainstream society. While it is clear that there is a complex and interrelated relationship between anti-science messages on social media and those who believe them, to date, only a handful of studies engaged directly with conspiracy theorists [51, e.g., 52], and none have targeted ex-conspiracy theorists to explore their journey back into mainstream society and what barriers they have experienced in this journey. This phenomenon, and the barriers experienced by those who wish to be rehabilitate into society, should be the focus of communicators of scientific information if as a society, we seek to overcome this dividing and disruptive force of scientific misinformation.

### The present study: Investigating the journey of ex-conspiracy theorists

Therefore, the present study employed a qualitative approach to capture the complex emotional and personal experiences of four ex-conspiracy theorists. By focusing on their narratives, we address a significant gap in the literature that often overlooks the lived experiences of individuals within conspiracy communities. The study explored what drew them to watch and believe in anti-science videos on YouTube, how these videos and the platform facilitated their journey into the conspiracy community, and what enabled them to eventually leave. Foreshadowing our results, semi-structured interviews revealed what made the anti-science messages persuasive and attractive; how participants’ own TIS was devalued; and how being part of an online community impacted their everyday thoughts and behaviours (e.g., health decisions, changes to friendship circles), sense of belonging, and personal circumstances. This examination sheds light on the persuasive tactics employed within anti-science communities promoted on YouTube and their implications for individuals’ behaviour, TIS, and social dynamics.

## Methods

### Approach to data collection

The study received ethical approval from Manchester Metropolitan University ethics committee (Ref Number: 25096). Before beginning the interview, CM checked that participants had read the participant information sheet and consent form that was sent via email for participants to look over. CM checked if they had any questions, and then audio recorded each participant’s verbal consent by reading through each item on the form and asking participants if they consent to each item. Data collection took place from the 24th of September 2020 to the 8th of October 2020. It involved a descriptive qualitative design [53] that was informed by an interpretivist framework [54]. Data about the experiences of four ex-conspiracy theorists was collected using semi-structured interviews, to enable the flexible collection of rich data from participants. Individual interviews were chosen as they allow participants to answer the questions in a personal experiential manner, and in-depth when appropriate [55]. A multifaceted approach was taken to address potential issues of trust and power within this study, a priority given the participants’ involvement in anti-science communities where mistrust of institutions like universities is prevalent. BH who led the recruitment process, spent several months observing activity within these communities and building rapport with potential participants who were invited to take part in the present study. This rapport was crucial for recruitment and was established through full transparency of the research process including clear communication about the aims of the study, what participation would entail, how data would be collected and anonymised and how confidentiality would be prioritised. BH personally introduced all participants to CM, who conducted the interviews. At the start of each interview, general questions were asked to build rapport such as ‘Tell me a little bit about yourself and how you first got into watching anti-scientific videos?’. CM applied a non-judgemental approach to interviewing, to ensure all participants felt comfortable sharing their views openly. To further enhance trust, participants were offered their transcripts, reassuring them that they could confirm the accuracy of their statements, and that no data had been altered. This transparency and rapport building process were key strategies in addressing mistrust and minimising power imbalances between researchers and participants.

### Participants

Purposeful sampling is a widely used technique in qualitative research to identify participants most relevant to the research aims and allowing researchers to make the best use of a limited and difficult-to-reach research pool [56]. The inclusion criteria required participants to have previously been active (e.g., interacted with other members, posted on social media sites, etc) members of an online conspiracy community, and subsequently decided to leave that community. Researcher BH identified suitable participants in the conspiracy community and contacted them via email or alternative electronic personal messaging services. This approach involved identifying the researcher to the participant, disclosing the study aims and inviting them to participate. Due to the unique and often stressful experiences of these participants within the conspiracy communities, many remain suspicious of established institutions, meaning an exchange of several messages occurred before consent for taking part in the study was gained. We assured participants of the importance of their anonymity throughout the entire research process. Therefore, we do not report participants’ age, gender, nationality, or other identifiable information. No incentives were provided for participation. BH identified nine potential participants, two immediately declined to participate due to suspicion over the process, three initially agreed to participate but then changed their mind for undisclosed reasons, and four participants agreed to participate in the study.

Qualitative research, particularly with a small sample size, aims to delve deeply into the experiences and perspectives of participants rather than providing statistically generalizable results. As Creswell and Poth [57] highlight, qualitative research is designed to explore the complexity and nuances of phenomena within a specific context, emphasizing the in-depth understanding of the studied phenomenon rather than broad generalization. This aligns with our methodological approach, which emphasizes the homogeneity of our sample to illuminate shared experiences within a distinct cultural or social context. Furthermore, Lincoln and Guba [58] introduced the concept of transferability, emphasizing the potential for findings from a qualitative study to be applicable or transferable to similar contexts or settings, rather than being broadly generalizable across populations. In this light, we acknowledge that our findings have relevance and applicability to similar settings or groups. Additionally, as Malterud, Siersma, and Guassora [59] suggest through their concept of ‘information power,’ our sample size is justified by the richness of information provided by participants. Information power indicates that the more information a sample holds that is relevant to the study, the fewer participants are needed, thus allowing us to focus on in-depth exploration rather than broad coverage. It is important to note that we include participants from three countries across different continents, but due to the sensitive nature of the participants’ responses and the volatility of their small communities, we have omitted this information for the sake of anonymity.

### Procedures

Interviews took place during the COVID-19 pandemic and were guided by a semi-structured interview schedule developed by BH, YB, and CM (see Table 1 for the specific questions posed to participants). These questions served as a guide, and if topics naturally arose during the conversations, they were not repeated. The aim was to explore participants’ experiences within the YouTube conspiracy community, including their initiation, engagement, and process of disengagement.

**Table 1:** Interview schedule.

Question number	Interview Questions	Purpose
1	Firstly, can you tell me a little bit about yourself and how you first got into watching anti-scientific videos?	Set the stage for personal narrative and rapport building, whilst establish context.
	Prompt – What were you initially searching on the internet or watching on YouTube for these videos to be recommended to you?	To explore the factors that led to initial exposure to anti-science content.
	Prompt – Why did you keep watching the videos, what kept you intrigued?	To understand sustained interest and engagement.
2	What role did watching YouTube videos paly in you adopting an anti-science perspective?	Directly addresses the core research question on influence.
3	For how long (days, months, years) and how frequently (daily, weekly, monthly) did you watch these videos for?	To understand the extent of exposure to anti-science content and viewing habits.
4	Which anti-science video would you say is the most influential on YouTube and why?	To identify key influencers and understand their impact.
5	Which other videos did you watch specifically and why were you drawn to them?	To explore the range of anti-science content consumed and the reasons for engagement.
	Prompt – Can you describe specifics about the videos that encouraged you to adopt an anti-science perspective?	To identify how content, narratives, and social influences in videos shape attitudes and behaviours
6	Which anti-science channels would you have recommended to someone who you wanted to know more?	To identify influential channels and key content that shaped perspectives. Also explores perceived credibility of sources within the community.
7	A credible source is generally agreed to be credible if it comes from the government or health professionals such as doctors or someone who is an expert in a certain field. How did the videos that you watched meet this criterion?	To examine participants’ perceptions of credibility and how they evaluated information.
	Prompt – How do you feel that the videos you watched could help to create doubt in established ‘credible sources’ such as universities and the government?	Explores techniques used in content to create distrust.
8	What other information about watching those videos would you say is important that has not yet been mentioned?	To allow participants to provide additional context or insights.
9	In what way did your anti-science perspective influence your life outside of YouTube? For example, how did it influence relationships with family and/or work colleagues?	To explore broader implications of anti-scientific beliefs on personal life, i.e., the real-world consequences of holding anti-science beliefs.
10	What examples can you give showing how your anti-science perspective encouraged you to engage in any behaviours that you may not have done before?	Explores behavioural changes resulting from beliefs
11	Do you feel religion plays a role in the anti-science communities? (if so, can you give any examples that may be personal to you or something you have seen?)	To explore the potential intersection between religion and anti-science beliefs.
12	Do you feel there is a connection between anti-science communities such as the anti-vaxxers, climate denial, flat earth? E.g. if you believe in one do you think people are more likely to believe in the other?	To examine the relationships and overlap between different anti-science communities.
13	What are your thoughts on anti-vaxxers? Did you ever feel persuaded by their argument? (explanation of why needed)	To explore participants’ views on specific anti-science movements.
	Prompts – What about the idea that there is a cure for cancer being hidden from us?	Probes familiar with conspiracy theories relating to health, whilst also considering motivation behind conspiracy beliefs.
		Prompt – Could you give insight into why some may believe this and what they think would be gained by keeping this from the public?
14	What caused you to start doubting the accuracy of these anti-science videos?	Explores the transition from belief to scepticism.
	Prompt – Would you describe this as a sudden or gradual process?	Seeks to understand the nature of the change in beliefs.
	Prompt – How did this influence your view on science?	Examines the broader implication of this shift in perspective.
15	When you first started to have doubts, what did you do?	To explore responses to emerging scepticism.
	Prompt – Did you speak to anyone in or out of the community or any anti-science YouTube channel providers? If so, what did that conversation look like?, If not, why did you not speak to anyone about it?	Investigates reactions and support-seeking behaviours when participants began to doubt their beliefs.
16	Why do you think there is mistrust between the anti-science community and established credible sources such as scientists, the government and researchers?	To understand the underlying reasons for mistrust and its implications.
	Prompt – Would you say that the mistrust in the community is real or do you think there could be other ulterior motives for people to promote an anti-science rhetoric and why?	Examines beliefs about community mistrust and motivations for promoting an anti-science rhetoric.
17	How do you think academics and universities could do better to deal with anti-science messages on YouTube?	To gather participants’ recommendations for addressing the spread of anti-science information.
18	Thank you so much for your time, is there anything else you would like to add before we finish?	To provide an opportunity for participants to share any additional thoughts or insights.

We designed the questions to be open-ended, encouraging participants to share how they began watching anti-science videos, which allowed for inductive exploration of their personal experiences. These were followed by more specific questions that delved into how YouTube shaped their beliefs and how they assessed the credibility of sources. This combination of open-ended and targeted questions enabled us to collect rich, detailed data to address key research objectives, while also allowing participants the flexibility to share their unique perspectives. At the end of each interview, participants were asked if they had anything further to add, ensuring no crucial information was missed.

### Data analysis

Data was transcribed by CM with the aid of Otter AI (https://otter.ai). CM, YB and BH manually corrected the transcriptions and BH and YB added gestures and comments where relevant. For transparency and to enrich the data collected, the final transcripts were offered to participants to see, and those who wished to do so, got their transcripts to review. This was one of the strategies used to ensure trust within our research process.

Analysis was supported by NVivo 12 software. Data analyses were guided using inductive, reflexive thematic analysis (RTA) as described by Braun and Clarke [60–62] who consider RTA as a reflection of the researcher’s interpretive analysis of the data [63]. Analysis was initiated by reading and re-reading each transcript carefully to become familiar with the data. Whilst doing this, annotations were added to highlight any primary insights of the data. This was followed by data coding, whereby each transcript was systematically coded, line-by-line, and sometimes with a few lines together to attain the context of the data being coded. All initial coding was performed by CM, BH compared and checked the correspondence between transcripts and initial themes for proposes of reflexivity, and YB checked coding by themes. The process of coding and theme development integrated descriptive (what the participants said) and interpretive elements (the researcher’s subjectivity to consider patterns not so obvious) (Braun and Clarke, 2006).

Following this, CM commenced theme development, with large patterns across the dataset being collated into meaningful themes. All common patterns of shared meaning, the central organising concept of TA [64], were grouped together to form themes and subthemes that were developed with the entire data set in mind [61]. Themes were shared with the research team (BH & YB) and were reviewed and refined several times as part of the analytical process, which required re-reading all codes that had been designated to a theme, to determine whether a meaningful pattern was evident. This was to ensure clear and concise themes with no overlapping. For an inductive, data-driven approach, a conscious effort has been made to avoid naming themes by categories and definitions from previous literature, whilst acknowledging the role the research has in the co-creation of themes [64].

A contextual constructionist perspective was taken, with the patterns of shared meaning identified in the text as socially produced, following the ideology that interpretation occurs with the influence of cultural values and meanings [65]. Any codes that could not be meaningfully integrated into a theme were grouped together and labelled ‘miscellaneous”. The writing up phase enabled for any themes that did not seem too ‘fit’ to be adjusted accordingly and the final thematic structure was achieved. All transcripts were re-read for a final time to ensure themes mapped onto study aims. Writing up the themes, involved selecting extracts from the data for each theme and subtheme and fitting this into a narrative that highlighted the views of the participants. CM discussed final themes with BH and YB.

Strictly to protect our participant’s anonymity, we excluded quotes that could potentially identify participants and redacted all identifiable information from quotes used in the paper. We did not return to participants to discuss the resultant themes, but those who asked to receive a copy of the results, were sent a link to the preprint version of the manuscript.

### Results and discussion

This study explores online conspiracies by interviewing four ex-conspiracy theorists about their experiences entering and exiting the conspiracy world. It examines what drew them into joining anti-science communities on YouTube and social media, how these platforms facilitated their adoption of conspiracy beliefs, and what prompted and encouraged them leave these online communities. Participants reported a range of conspiracy beliefs, including general distrust of science, 9/11 terror attacks, flat Earth, mass shootings, COVID and vaccine misinformation, and scepticism toward conventional cancer treatments. The study also identified techniques that particularly persuasive when used by non-experts on social media, and what information was effective or ineffective in guiding participants back into science-based views. Using Reflexive Thematic Analysis (RTA), we identified four key themes and eight subthemes, summarized in Table 2. We then use this information to inform the implementation of empathetic interventions to address those who hold anti-scientific misinformation and conspiracy beliefs.

**Table 2:** Themes and subthemes.

Themes	1	2	3	4
	Gateway into the Echo Chamber	The Puzzle of Scientific Illiteracy	The Pull and Power of the Conspiracy Community	Escaping the Echo Chamber
***Subthemes***	1.1. Triggers of Conspirational Beliefs: Loneliness and Life Events	2.1. Lost in Translation: From Illiteracy to Illusion of Expertise 2.2. A Web of Mistrust: Tactics to Discredit Scientific Sources	3.1. From Identity to Authority: The Dynamics of Conspiracy Communities 3.2. The Power of Mantras: Repeat, Repeat, Repeat 3.3. Conspiratorial Watchdogs: Policing the Gates of the Community	4.1. Clash of Narratives and Seeds of Doubt: The Exit out of Conspiracy 4.2. Exit Wounds: Navigating Life after the Echo Chamber

## Theme 1:–Gateway into the echo chamber

This theme captures how participants first became immersed in conspiracy beliefs, detailing the experiences and influences that drew them into, and sustained their involvement in, virtual echo chambers. Participants recognized the growing impact of the videos on their evolving beliefs.

### Subtheme 1.1. Triggers of conspiratorial beliefs: Loneliness and life events

All participants discussed their observation that significant life events often coincided with their own and other individuals’ discovery of conspiracy videos online.

*“The common theme that I see with everyone is that there was something going on with their life, like they just broken up from a marriage or they just had a bad boyfriend experience or, you know’… one of the parents have just passed away or … they we’re just becoming a parent for the first time … it always felt like there was something significant that happened in their life and emotional sort of significance, um, was miraculously when they found... a video.”[P1]*

These moments of personal vulnerability often acted as ‘tipping points’, making individuals more receptive to alternative narratives offered by conspiracy theories. Participants also described how feelings of isolation and loneliness increased their engagement with these communities:

*“I just found myself being in quite an isolated situation socially um, sort of, probably from my choosing, I’ve withdrawn from society a little bit. So, my interaction was really these live videos.” [P1]*

*“Because fundamentally, they’re lonely. I was lonely as well, exactly the same for me, you know I might have slightly different perspective, but fundamentally lonely, I was lonely at the time.” [P4]*

These accounts align with Douglas et al. [66] who proposed that conspiracy beliefs often stem from epistemic (the understanding of one’s environment), existential (being in control of one’s environment), and social motivations (the maintenance of positive images of the self and the wider social group). Similarly, Tetlock [67] noted that individuals have a desire for both personal and collective control over their environment, becoming more prone to conspiratorial thinking and endorsing superstitious beliefs during uncertain times (e.g., COVID pandemic), such as significant life events [68]. Participants responses also echo research showing that feelings of powerlessness and anxiety are associated with the increasing likelihood of adopting conspiracy beliefs [69,70].

A recurring theme was the interplay between significant life events, loneliness, and the gradual immersion into conspiracy beliefs. Participants noted that major negative events often provided more time to engage with conspiratorial videos and communities on YouTube, for example:

*“I think relevant factors might have been that I had a lot of family members die … So yeah, I was just, you know, I wasn’t at work, um, for some of when I first started watching. So, I had a lot of spare time and started watching YouTube and yeah, and it just went from there.” [P4]*

Phrases such as ‘it just went from there’ suggests the initial engagement with YouTube videos evolved overtime into a more sustained and immersive experience, which eventually would lead to some acceptance and adoption of the conspiratorial beliefs. As such, free time, coupled with emotional vulnerability facilitated deeper involvement with conspiracy-related content, which for some had an almost addictive quality:

*“I didn’t just become a flat earther, these videos I was watching them for months in and out on my feed. I remember saying…I think YouTube’s turning me into a flat earther.” [P4]*

*“It became enjoyable to watch these videos and feeling like I was learning the “truth” from them gave them almost an addicting quality.” [P3]*

Participants recognized the influence of algorithms in shaping the content they were exposed to and how this contributed to their deepening engagement with conspiracy beliefs.

*“The issue was I wasn’t watching or getting recommended any actual scientific videos, I was in an echo chamber at this stage and also not actively seeking any videos about real science until much later.” [P1]*

*So, I was aware of that, and it [YouTube] kept pushing these [conspiracy-related] videos at me.” [P4]*

This illustrates how negative life events, loneliness, and YouTube’s algorithms combine to create a conducive environment for the spread of misinformation. Research indicates that false news spreads faster than truthful information. Vosoughi, Roy, and Aral [32] conducted a systematic analysis of around 126,000 tweets from approximately 3 million people, revealing that false statements were re-posted more quickly, reached larger audiences, and were more emotionally engaging than true news stories. Similarly, Bora et al. [28] found that YouTube viewers were more likely to share and like videos containing false information compared to accurate ones. This demonstrates a unique appeal of misinformation, prompting users to share it rapidly. Additionally, evidence suggests that YouTube’s algorithms amplify extremism, potentially steering users toward greater polarization over time [71–73]. This indicates that YouTube users are not primarily discovering misinformation through their own searches but rather they are being directed to these videos by the platform itself [74], a phenomenon our participants were only too aware of yet unable to prevent. While the transformation into conspiracy adherents is not solely attributable to algorithmic recommendation systems utilized by platforms like YouTube, it is evident from the participants’ narratives that these systems play a significant role in shaping echo chambers and strengthening and intensifying pre-existing conspiracy beliefs.

## Theme 2: The Puzzle of Scientific Illiteracy

This theme explores the issues of scientific illiteracy of those within the conspiracy community and subsequent susceptibility for belief in conspiracy theories. Two subthemes were identified, both highlight the role of scientific illiteracy in the facilitation and justification of, participants’ collective belief in conspiracy theories.

### Subtheme 2.1. Lost in Translation: From Illiteracy to Illusion of Expertise

Participants noted that individuals in conspiracy communities often had a limited understanding of scientific concepts and methods, largely due to a lack of higher or science education. This was seen as a major factor in their acceptance of the information shared within these communities.

*“… I think it comes down to education … I think, because science is one of those disciplines that, like I said, unless you’ve got a good understanding, or a foundation, at least, it’s very difficult to understand something like um quantum physics … even for the average Joe.” [P1]*

The community’s limited scientific education and understanding made them more receptive to the information shared. In hindsight, individuals recognized they lacked the knowledge and critical thinking skills needed to assess and challenge conspiratorial claims. This is consistent with research showing that lower educational attainment is linked to a greater likelihood of adopting conspiratorial beliefs and playing a key role in fostering the acceptance of anti-science information [75,76]. For example, participants recognized that their lack of understanding of the scientific process made conspiracy theories more appealing, as these offered simple and accessible explanations for complex phenomena.

*“… a few of the flat earthers admitted … they’ve never read a book in the[ir] whole entire life and they were adults … they just had a very limited education …. they were happy just watching these YouTube videos … they’re really not interested in … researching or reading anything that’s past two sentences. So, they’re going to stay in that echo chamber.” [P1]*

The observation that lack of understanding of scientific processes can make conspiracy theories appealing by offering simple and accessible explanations for complex phenomena, is consistent with Klein and Robinson [77] who observed that social media allows non-experts to share simple yet incorrect information with others who similarly lack scientific knowledge. Our participants noted that this serves to reinforce core beliefs of the community, as like-minded individuals validated each other, strengthening social cohesion and entrenching conspiratorial thinking.

Despite lacking a research background, the community strongly promotes the mantra of “Do your own research,” which typically means watching more YouTube videos. This underscores their reliance on online platforms, particularly YouTube, as a primary source of information, fostering a false sense of expertise.

*“You know, a lot of people will say …’ I’ve done my research’,’ do your research’, when really that involves … watching YouTube videos, or reading news articles.” [P3]*

Participants did note moments when they realized the shortcomings of ‘researching on YouTube’ compared to actual scientific research, emphasizing the role of access to scientific literature and knowledge in changing attitudes.

*“But figuring out that you can actually go to scientific journals and read work that involves data, data analysis and actual experiments done by professionals who have been peer reviewed all that stuff. Once I learned all that my perspective on how to learn, completely changed.” [P3]*

This shift in perspective demonstrates how access to information, combined with understanding of the scientific process can transform how individuals seek and interpret information [78]. Scientific literacy refers to the ability to comprehend science articles in popular media, discuss their societal implications, and critically assess the quality of information based on its sources and data [79]. This skill, not only enhances critical thinking but can also protect individuals, even those prone to conspiracy beliefs, from the influence of non-scientific YouTube videos [46]. Participants reported they and others within the communities were lacking sufficient scientific literacy, including critical thinking skills, to identify the falsehoods they encountered within the conspiracy community and on YouTube videos. These findings are supported by Luo and Jia [80], who suggested that lower scientific literacy is linked to a higher likelihood of adopting conspiratorial beliefs.

Participants reported that they had come across debunking videos and opposing information. While some found it useful, others felt it was hard to understand or that it didn’t address their concerns at the time. This could suggest that they may have been more likely to focus on information that supported their beliefs at the time, or that the videos were not sufficiently accessible to those with low scientific literacy or low trust in authority.

*“A lot of the content that I would watch was conspiracy related and there wasn’t a lot of debunking videos that I came across at that time, at least. There were a few, but they weren’t, they just weren’t attractive to me at the time, because it was always like, you know, a debate. I remember…for example, debate between the guys who made Loose Change and um two people from the Popular Mechanics Magazine … But what I remember was they weren’t talking in a way that, to me, debunks the points that I felt were most convincing in the 9/11 conspiracy.” [P3]*

Additionally, some participants reported feeling that those in the debunking videos were laughing at them, which negated their purpose to inform and debunk anti-scientific information.

*“What used to frustrate me a bit as well with the flat earth debunking is they would just laugh at them.” [P4]*

This suggests that using mockery or laughter for debunking conspiracy theories is ineffective. Perhaps more substantive and constructive debunking methods would better facilitate belief revision. Support for the use of critical thinking as an intervention to counter conspiracy beliefs is echoed by O’Mahoney et al. [78], who highlighted the importance of fostering analytical thinking skills as amongst the most effective interventions for countering conspiracy beliefs. Rational arguments, especially those presented without personal attacks, seem less threatening to conspiracy theorists than ridicule, which may provoke defensiveness. The mechanism of change may involve the activation of ‘rationality heuristics,’ which are more likely to be triggered by non-confrontational, logically structured arguments that present facts and figures [81].

### Subtheme 2.2. A web of mistrust: tactics to discredit scientific sources

Deceptive tactics such as ‘cherry picking’ were reportedly employed often by those within the conspiracy community. This tactic of selecting specific segments of information and crafting a narrative that aligns with the preconceived beliefs was used to support conspiracy claims. This is consistent with research showing that misinformation often hinges on the selective presentation of data, where misrepresentations serve to create doubt regarding the credibility of scientific information and associated scientific institutions [82]. As noted by our participants, thus:

*“I could sort of sense that, hang on, surely no one from the university has said this? So, it was sort of like the fact that I could go to citations that they gave, and I actually read the full citation and realised hang on, they’re cherry picking.” [P1]*

*“… when it comes to the doubt in the, you know, breaking apart what is established… it’s often done by cherry picking, cherry picking certain segments of a lecture, and then creating your own narrative of that without giving without giving the whole truth um, and I think that that is deceptive in its own right…deception is the tactic that they use to … blur the lines between what the science actually says and what they say.” [P2]*

*“One requirement, um, in believing these things is doubting the official sources: the scientific community, the government, whatever … it is THAT you’re doubting. That’s a requirement, right? Because I mean, for example, flat earthers, they have to deny that NASA is faking all those pictures of space, or else, they can’t have any of their beliefs.” [P3]*

It appears that conspiracists maintain their alternative beliefs by casting doubt on the legitimacy of official sources, viewing them as part of a broader deception or cover-up. Bunker [83] described this as the “digital destruction” of truth, where echo chambers on social media systematically erode shared realities and factual understanding. Over time, this scepticism deepens, leading individuals to distrust information from official sources, even when they recognize that parts of it may be true. Over time, this growing distrust not only sustains conspiracy beliefs but also strengthens the bond within these communities, as members collectively reject official narratives. This shared scepticism often extends to government and scientific institutions, as one participant explains:

*“I think it’s because they set it up right to be that you can’t trust the government and … if you can’t trust the government, you can’t trust scientists, because scientists will work for the government. Um and it starts with NASA, NASA is out there getting, you know, $55 million a day, or whatever number they throw out there um and all these people know that it’s just the lie and it’s all CGI.” [P1]*

This subtheme reiterates the interconnectedness between ‘cherry picking’ mis/information, the use of social media to amplify misinformation, and the erosion in trust in scientific and official institutions. By challenging the integrity and motivations of government and scientific institutions, the conspiracy community can create their own narrative. These findings are supported by Khalaf and Sheata [84] who noted that trust in official sources moderated the relationship between exposure to misinformation and beliefs that this information was true. Indeed, our participants reported that information provided by such organisations was perceived to be untrustworthy, unreliable, and manipulated as part of a larger conspiracy to hide information from the public. In turn, this growing scepticism was used within the echo chamber to maintain the illusion of expertise (i.e., ‘we know better’) in the community (Subtheme 2.1).

## Theme 3: The pull and power of the conspiracy community

Within the realm of conspiracy beliefs, this study highlighted that the power of community should not be underestimated. Community was a dominant theme across interviews and participants made it clear that it was the community that enabled conspiracies to thrive within the echo chambers. We also observed that community strengthened individuals’ feelings of belonging and identity as their alternative views were accepted. The need for a community among our participants, possibly due to significant life events they had experienced, was a ‘draw’ for participants into the conspiracy world. Some reported that these experiences replaced missing aspects in their ‘real-life’ (as described in Theme 1). However, the need for community also served as a barrier for participants challenging or leaving the community. Three subthemes were identified.

### Subtheme 3.1. From identity to authority: The dynamics of conspiracy communities

Activity and interactions of members within the community were described as extensive and at times an all-consuming ‘hive of activity’, with strong personal connections and sense of purpose. While the online platforms serve as the initial gathering place, participants described how the community extends beyond the digital realms, which further deepened the connections among community members.

*“The community online, or the live streams that you see is one thing. But offline, the flat earth is this hive of activity... those people... are still connected... looking at videos coming up from ‘the globe’[Note; the name from within the community for the ‘scientific community that believes in a ‘round’ rather than ‘flat’ earth], looking at videos coming out from the government, finding documents... it’s this hive of activity just all the time... it’s almost like it’s coming from a top-down authority to stamp out certain narratives... and it never sleeps.” [P2]*

This intense interaction strengthens bonds between members, fostering a strong sense of purpose and belonging. Greve et al. [85] noted that the spread of conspiracies theories on social media was not always driven by biased algorithms *per se*. Rather, those who share their conspiratorial ideas with the like-minded group, become part of a larger community, one that reinforces and amplifies their beliefs. Once a part of this group, participants reported that it provided them with the sense of belonging and status that they lacked and enjoyed:

*“…Yeah, and that gave you a real sense of belonging.” [P1]*

*“Now I’m somebody, now I’m just not a nobody. And then at the end of it inside, that is a superiority complex, complex. I’m above all those PhDs. I’m above all those, you know, people, and I think that’s the that’s the trick. That’s the best the hook inside the flat earth.” [P2]*

By belonging to a group of “the enlightened” who possess secret knowledge and insights, the ingroup was able to create a sense of superiority and empowerment [86]. Their ‘secret knowledge’ extended to other aspects of science scepticism:

*“… basically, just this idea that there’s some hidden cancer cure that is being kept from us.” [P3]*

*“I’m not going to wear a mask. I’m not going to get the vaccine. This is just scientists trying to control us.” [P1]*

This new ‘knowledge’ empowered the participants, and gave them a sense of superiority, enabling them to overcome their own feelings of inadequacy:

*“As someone who never really excelled in grade school, believing conspiracy theories made me feel intelligent and that I had access to knowledge that others didn’t.” [P3]*

Previous work highlighted that feelings of exclusion from mainstream society, coupled with personal interactions within the conspiracy group, play a pivotal role in drawing people deeper into these ideologies [87]. Being part of the community allowed participants to feel valued and important, and membership of the community became an important aspect of their identify:

*“I remember … making it kind of part of my identity, I would, I would buy their DVDs, and um… DVDs, a couple of stickers, and I put their stickers on my computer.” [P3]*

This quote demonstrates how the group’s ideology merges with the individual’s sense of self, blurring the line between personal beliefs and those of the collective. Social identity theory supports this, suggesting that the desire for strong group membership significantly shapes behaviours and identity [88]. The sense of belonging and perceived access to exclusive knowledge further enhances self-esteem and solidifies one’s identity within the group [89]. This creates a self-reinforcing loop: a weak personal identity drives the desire to belong to a group that offers a stronger sense of identity, then as individuals become more involved, their identification with the group intensifies, which further solidifies their commitment to its ideology [90].

Participants described how being part of the conspiracy world created an ‘extended network, as conspiracy communities often reached out to other online groups, fostering a sense of belonging to a larger society and potentially serving as a recruitment ground:

*“It was never just one conspiracy theory that I believed it was always multiple.”[P3]*

*“I think what the smart flat earthers are working out is that you know they’re losing numbers, so how do we get them back? Or how do we get new people or new blood coming. Oh Wow, okay, cool Next sort of topic. 5G’s killing everyone, oh the coronavirus isn’t real, oh don’t vaccinate children. You know now with the vaccine that you know; we’re all waiting for the coronavirus. Oh, God, no, we’re not getting the vaccine, you know.” [P1]*

These insights highlight the community’s unwavering commitment, with social media acting as a cross-platform hub for the constant exchange of (false) ideas, reinforcing both the shared beliefs and their sense of authority. [91,92].

### Subtheme 3.2. The power of mantras: Repeat, repeat, repeat

Within the conspiracy community, there is a tendency to repeat arguments in a style akin to cult-like ‘chanting’. The repetition of mantras, memes and arguments were described by participants as central to the community even if arguments have already been refuted.

*“…these are the channels that people will repeat from them verbatim, you know, and parrot argument that’s already over and already destroyed, so to speak.” [P2]*

According to Béna et al. [93], people are more likely to believe conspiracy theories they have encountered repeatedly, regardless of the actual validity of the statements. This s known as the “truth effect,” where repeated exposure enhances the familiarity of a statement, making it seem more credible. The repetition of such mantras increases their persuasive power, and the reductionist explanations (e.g., the flatness of the horizon or the absence of perceived motion) can appear ‘reasonable’:

*“All of this stuff sounds right, you know, like, oh, but I look out on the horizon, and everything looks flat. I can’t feel myself spinning at 1000 miles an hour. So, I think all these sort of catchphrases that … they all use and sometimes in different ways, but it’s all sort of the same propaganda” [P1]*

The use of the word “catchphrases” in the above quote suggests that these ideas are often repeated in a way that reinforces a specific narrative within the community. This simplification of complex information into easily repeatable soundbites is in accord with research showing that repeated exposure and ease of recall provide individuals with a sense of familiarity, which in turn makes the information (irrespective of its actual truth) more believable [94,95]. As a result, participants noted that the community tends to reward those who conform and repeat these beliefs:

*“…for just repeating every day the same thing you know, with a panel that said yes to everything.” [P2]*

Such repetition was socially rewarding within the community, highlighting the power of the online conspiracy community to create an instant sense of camaraderie and connection.

*“… it’s quite powerful that if you are new to say, flat earth … and you’ve gone on a stream and it’s all these people in the chat live chat on the side and you could type in something … [like] ‘stupid globs’ or, you know, ‘the earth’s flat’, whatever you type one of their silly memes in that they’ve got at the moment, all of a sudden you have loads of friends. No reason other than you just agree.” [P4]*

Participants often described that recognition and social connection within the community were achieved by repeating shared beliefs and phrases, which in many cases led to building their own social channels and gaining a form of “fame” within the community. This repetition not only strengthened group identity but also created an atmosphere of validation and authority, making dissent increasingly unlikely. Research on echo chambers suggested that repeated interactions within a closed community reinforces shared beliefs while insulating members from opposing views [93]. The power of repeating mantras also contributes to cognitive biases, such as confirmation bias, where individuals selectively seek and focus on information that aligns with their existing beliefs [96]. This behaviour, like the “cherry picking” observed in the previous theme (2.2), serves to further entrench community ideology and reduce openness to contradictory information.

### Subtheme 3.3. conspiratorial watchdogs: Policing the gates of the community

This subtheme explores the dynamics of strong internal policing within the conspiracy community. Participants reported an unspoken established system of monitoring and regulating what is ‘acceptable’ within the community, which helped maintain ideological conformity. This served to delineate clear “in” and “out” group boundaries and it appears to be in the core of ensuring group loyalty.

*“The flat earth community is so quick to turn on their own and I think that’s the biggest lesson. When you’re with them, they’ll do everything, invite you to the house, they want you to meet their kids … introduce you to the family, but the moment you’re against them, you are lower than dirt. So, you are just scum, everything you did and everything you were is [now] nothing.” [P2]*

This highlights the high value the community places on conformity and loyalty to their shared beliefs. Group censorship and ‘gatekeeping’ of ideas were used as a mechanism to safeguard shared beliefs and prevent divergence from the accepted narratives [97]. Opposing the group’s ideas was not tolerated, and those who deviated from the established narrative were ostracised and devalued:

*“I learned very early on that … the flat earth community was such a, such a volatile … place when it when it comes to saying the wrong thing and being now one of them or not, [being] a real flat earther or not, [being] a … real conspiracist or a government agent.” [P2]*

Participants outlined a drastic shift in the way they were treated by the community-from being warmly welcomed into the community to being viewed as ‘scum’. A tactic that is often utilized to differing degrees in various cults [98,99]. This further highlights the community’s response to dissent and their cult like manner, which limits members’ ability to question the dominant view of the community:

*“… if you ask a question, you’ll be shunned if it’s not the right question... it’s no different to a cult, in that sense … there’s no challenging whoever the priests are of that particular group.” [P4]*

Participants described that keeping the boundaries between ‘us and ‘them’ extended to trolling (and condemning) those outside the community, which also served to build a shared sense of camaraderie within the community:

*“… it would be basically just trolling them, like witty comebacks that would sort of make the rest of the chat [Note: those in the conspiracy group] laugh.” [P1]*

Trolling also served as a strong deterrent for individuals wanting to leave the community. Participants recounted toxic practices, including harassment, and contacting individuals’ employers, which serve as deterrents to expressing doubt or attempting to exit the community:

*“So, you know, they’re a nasty community … they have rung up people’s employers and like, like I said, its people’s real life […]. So, people see that and … think, well, I’ll either just bow out without anyone noticing and not saying anything because of the repercussions” [P1]*

*“… [I] turned on my tablet after I, after maybe, I don’t know, maybe six or seven months [after leaving the community], I had 128 missed calls from people in the group, you know, wondering where I’d gone, what I’d done … and the hate I received from these people. “F U you actually gone back to the [globe] bandwagon.” [P2]*

Overall, this theme illustrates the pervasive culture of intimidation within conspiracy communities, particularly against those who challenge or reject community doctrines. Participants described strict enforcement of group norms and harsh consequences for dissent. This is consistent with previous research on gatekeeping and controlling behaviours in such groups [100]. Policing serves to maintain unity, reinforces collective identity, and discourages divergence. Fear of “life after” the community was a significant barrier for many participants, a theme often seen in cult dynamics [101], with similarities between conspiracy groups and cults being evident [102–105].

## Theme 4: Escaping the echo chamber

This theme explores the journey of individuals who have managed to escape the echo chamber, despite the few paths available for those who wish to leave the strong-knitted conspiracy community. This theme is constructed of two subthemes.

### Subtheme 4.1. Clash of narratives and seeds of scepticism: The exit out of conspiracy

A significant precursor to leaving the community, reported by all participants, was that they never fully believed (perhaps retrospectively) every aspect of the community’s doctrine:

*“So, there were certain things that I never brought in, fully into, right. So, there was still a part of me that didn’t believe the whole thing.” [P1]*

*“… I never completely, um, took everything that was being told to me 100%.” [P2]*

*“I would get a little nervous because I knew that my argument wasn’t good on some level.” [P3]*

*“I also don’t think I ever really fully believed all that stuff….” [P4]*

This is important, as it suggests that every member of these online communities has a little doubt, and those who seek to reform conspiracists back, need only find and reveal that doubt, rather than plant it. Additionally, in a manner like the ‘route in’, which was described by participants as part of a personal journey following a significant event in their life, the way out was also often marked by being confronted with evidence that contradicted knowledge or skills that participants considered central to their life or identity.

*[Note: P1 described being asked to support the community using their professional expertise, and then said:] “I just started to think, hang on a second, I cannot condone this, like, flat earth is one thing. But now all of a sudden, this is like my professional world, my real life … to me, it was crossing a line of ethics that I just wasn’t comfortable with … I just tore it apart on a professional level. And that’s really when they turned nasty.” [P1]*

The deep sense of ethical conflict prompted and empowered P1 to challenge the community’s beliefs. Findings of a systematic review suggest that a personal ethical crisis often plays a significant role in pushing individuals to reconsider their affiliation with conspiracy groups [106]. However, another participant described a more gradual process to their exit:

*“So, it wasn’t an all at once … the gears started turning a little bit and … I started slowly, throwing out arguments until it reached a tipping point where it was like okay … I can’t believe this anymore.” [P3]*

P3’s journey out of the conspiracy community begun through a personal encounter with an anti-conspiracy activist, which instigated their rehabilitation process:

*“… the person who I had that really long conversation with … was doing a thing on YouTube, where he would say … what is the one thing that most convinces you that 9/11 is an inside job? Let me know and I will debunk it.” [P3]*

The role of a significant “outsider” as a catalyst for change aligns with findings that emphasize the importance of peer influence in both fostering engagement and facilitating disengagement. Such relationships have been shown to be crucial when leaving cult-like environments [ [101]. Peer interactions that present alternative viewpoints and challenge existing norms are effective in prompting individuals to reconsider their beliefs and eventually disengage from extremist ideologies [107]. This is consistent with research identifying social support as a key resilience factor against radicalization, abuse, mental health issues, and transitions in gender identity [108–111].

Participants also reported that coming across evidence that contradicted the doctrine of the community was a key motivator for them to leave the conspiracy community. However, a significant hurdle was the difficulty in accessing and accepting accurate information while still “under the spell” of conspiratorial beliefs:

*“… when you’re sort of investigating, you come across a lot of debunk videos. …. But when I was in that community, there’s no way that I would have watched any debunking video[s], because they’re all liars.” [P1]*

Participants noted that following their own specific ‘tipping point’, they experienced a change in perspective as they became more open-minded to diverse viewpoints that presented evidence contradicting the conspiracy theories embraced by them and their community. While participants may have got to their tipping point through different paths, their experience emphasises the importance of having easily accessible quality, evidence-based videos that encourage viewers to employ criticality and present a valid and attractive alternative to both condensing videos and those promoting conspiratorial beliefs [78].

### Subtheme 4.2. Exit wounds: Navigating life after the echo chamber

All participants were aware of how difficult other members had found the process of leaving the community before them.

*“I’ve seen other flat earthers try to leave and then they come back, you know, because there was monetary gain … or the community was there and then they feel loneliness, I guess.” [P2]*

This excerpt relates to Subtheme 3.3, “Conspiratorial Watchdogs: Policing the Gates of the Community,” where participants discussed the negative aspects of their communities and the potential repercussions of leaving, which ultimately influenced (and delayed) their decision to exit the group. Leaving conspiracy communities or cult-like groups is particularly challenging due to both internalized beliefs and the severe backlash from members. The worry about the ‘life after’ was previously reported to be a barrier of leaving cults [101], and the similarities between conspiracy groups and cults is evident in this respect [102–105].

*“Basically, there are about three trolls that have been in the community … that know everyone in the community … and … everyone in the community, they know what they’ve done in the past. So, it was really talking to them. And they encouraged me to just like, (identifiable information removed) disappear, because these guys will come after you, as long as you’re out there. And so that’s when I sort of just decided to disappear.” [P1]*

This illustrates the extent of internal surveillance within conspiracy communities and the threats faced by members attempting to leave. Such practices, which include intimidation and doxing (i.e., revealing personal information), aligns with studies highlighting how conspiracy communities actively discourage leaving through fear of retaliation and exposure, making the exit more challenging [87].

Participants described breaking their connection with the community in a ‘cold turkey’ manner.

*“… that was my exit strategy … I left just that … [and] never returned to the voice you know … deleted the group from … my tablet that was it. Never want to deal with you again type thing.” [P2]*

Due to the severity of the backlash and the manner that the community imbues a strong sense of belonging in participants, it is not surprising that when leaving the community, participants experienced a fundamental change in their social identity.

*“I think the backlash is so severe, that it’s not just about … changing your mind. It’s about saying, saying goodbye to all those things you’ve agreed to laugh, what you know, you spent … countless hours with these people agreeing with them, became better friends because of your things you agree with and the idea of confronting that … is harder than just leaving, just disappearing into obscurity.” [P2]*

This highlights that despite being aware of the somewhat cult-like negative aspects of the community, it is still a daunting task to leave. It requires a change of views and opinions, as well as relinquishing the camaraderie, and the feelings of importance and superiority that came from aligning with the community’s beliefs. This theme captures the emotional and social vacancy that results from leaving the conspiracy community [see 112].

## Empathetic interventions for addressing conspiracy beliefs

Per the qualitative research principle of transferability [113], our findings have valuable implications for science education and interventions aimed at individuals vulnerable to scientific misinformation on YouTube and other online spaces. Our approach prioritizes prevention, which is in to the outstanding work of Engel, Phadke, and Mitra [114] which emphasized recovery and support, particularly through online recovery communities that aid disengagement and address the mental health challenges associated with conspiracy involvement. By integrating insights from both studies, we can develop a more holistic understanding of the processes of engagement and disengagement, offering a comprehensive framework that encompasses both prevention and recovery. Therefore, guided by our findings, we propose three key intervention strategies: (a) enhancing scientific literacy among YouTube users, (b) recognizing the influence of personal experiences, and (c) implementing empathetic, platform-specific interventions. In addition, we highlight some potential pitfalls that need to be considered in each of these areas.

## Enhancing scientific literacy among youtube users

Scientific illiteracy significantly contributes to the susceptibility to unfounded conspiracy theories, especially on platforms like YouTube, where misinformation proliferates rapidly. While traditional efforts have concentrated on formal education settings, there is a pressing need to reach adult audiences who consume content on YouTube. Our participants reported that scientific information is often presented in a manner that is difficult to understand and sometimes condescending, particularly when addressing conspiracy-related content.

To bridge this gap, content creators and science communicators should focus on producing accessible, engaging, and respectful content tailored for the YouTube audience. They should consider designing content that overcomes barriers often seen in those with a susceptibility to believing conspiracy content. This includes creating videos that simplify complex scientific concepts without compromising accuracy, using relatable examples, and fostering an inclusive tone that invites curiosity rather than alienation. Such an approach can demystify science and empower users to critically evaluate the content they encounter on the platform. Recent studies have demonstrated that brief scientific literacy interventions, especially those focusing on critical thinking skills, can effectively reduce belief in conspiracy theories [115].

One significant pitfall to be aware of in this context is the potential for direct counterarguments to reinforce existing beliefs, a phenomenon known as the ‘backfire effect’ [116]. When individuals are presented with information that contradicts their deeply held beliefs, they may become defensive, leading to a strengthening of those beliefs rather than a re-evaluation. This effect underscores the importance of employing strategies that are not confrontational but instead encourage self-reflection and critical thinking. For instance, engaging individuals in dialogues that prompt them to question the foundations of their beliefs is potentially more effective than outright refutation [117].

## The role of personal experience in shaping beliefs

Our study uniquely identified that deeply emotional and personal experiences significantly influence participants’ susceptibility to conspiracy theories. Individuals often turn to YouTube communities seeking validation for their experiences and emotions, which can lead them into echo chambers that reinforce conspiratorial thinking. This behaviour aligns with findings that personal experiences and emotional states play a crucial role in the formation and reinforcement of conspiracy beliefs [118,119].

Interventions should, therefore, consider the personal and emotional contexts that drive individuals toward these communities. This involves creating supportive online spaces where users can share their experiences and access evidence-based information that resonates with their personal narratives. Collaborations between mental health professionals and content creators could facilitate the development of content addressing underlying emotional needs, such as loneliness or mistrust, which we observed often underpin the entry into and adoption of conspiracy beliefs. Research indicates that emotional factors, including anger and feelings of powerlessness, can significantly influence belief in conspiracy theories [119].

Our study emphasizes the need to focus on personal and emotional factors, perhaps placing less emphasis on factual rebuttals when designing suitable interventions. This approach is supported by evidence suggesting that addressing the emotional and psychological underpinnings of conspiracy beliefs can be more effective than solely providing information [120].

While creating supportive online spaces tailored to individuals’ emotional experiences can foster trust, it also risks reinforcing insular communities that validate, rather than challenge, conspiracy beliefs. Emotional validation, if not carefully balanced with exposure to corrective information, may inadvertently deepen belief entrenchment [121]. Additionally, the strong personal investment in these beliefs means that individuals may resist interventions perceived as undermining their lived experiences [122,123]. To mitigate this, interventions must integrate both emotional support and gentle exposure to alternative perspectives, fostering cognitive flexibility without triggering defensive reactions.

## Empathetic, platform-specific interventions

Engaging with individuals entrenched in YouTube-based conspiracy communities requires a compassionate and platform-specific approach. Efforts to challenge these beliefs should prioritize empathy and understanding, recognizing the complex motivations behind them.

One effective strategy is the application of nudge theory within the YouTube environment. This involves subtle modifications to the platform’s architecture to promote exposure to credible, science-based content. Nudge theory suggests that modifying the online environment can subtly influence behaviour without restricting choices [123]. Presenting scientific information online aligns with this theory, as it helps shape users’ decision-making processes in a more informed direction. Nudge theory proposes that positive and adaptive behaviour change can be achieved through strategic and intentional modifications to the environment in which decisions are made [124–126]. For instance, YouTube could adjust its recommendation algorithms to suggest emotionally and personally directed interventions alongside conspiracy-related videos, providing users with alternative perspectives without restricting their choices. This approach aligns with findings that modifying online environments can influence behaviour positively without limiting freedom [127], which is often a key fear of those in conspiracy groups or exiting them. However, a critical consideration of the nudge theory approach is that it may not effectively reach or influence individuals who are deeply entrenched in their beliefs or who actively avoid scientific information. These individuals often exist within echo chambers, where they are primarily exposed to information that reinforces their views, making it difficult for nudges to penetrate these insular communities [128]. Additionally, people tend to seek out information that confirms their preexisting beliefs and ignore or dismiss information that contradicts them, which could limit the effectiveness of online nudges. By acknowledging these limitations, we can better understand the challenges of implementing nudge-based interventions and future research can explore complementary strategies to enhance their effectiveness.

Additionally, fostering open dialogues through comment sections and live discussions can create opportunities for respectful engagement. Training content moderators and creators in empathetic communication techniques can help de-escalate confrontations and guide users toward critical self-reflection. Research suggests that empathetic engagement, rather than confrontational fact-checking, is more effective in encouraging individuals to reassess their beliefs [129]. While empathetic engagement is crucial, there is a delicate balance between validating individuals’ experiences and providing corrective information. Overemphasis on empathy without adequately addressing misinformation can lead to the perpetuation of false beliefs. Conversely, focusing solely on factual correction without considering the emotional and psychological dimensions may result in resistance. Therefore, interventions should integrate empathetic communication with clear, accurate information to facilitate meaningful belief revision [129].

In summary, addressing conspiracy beliefs propagated through YouTube necessitates interventions that are scientifically informative, personally resonant, and empathetically delivered. By tailoring strategies to the unique dynamics of YouTube communities, we can more effectively counteract the spread of misinformation and support individuals in critically evaluating their beliefs. In addition, while our study is focused on ex-conspiracy theorists, the insights gained can offer valuable perspectives for other sensitive populations. For instance, individuals leaving religious communities, extremist groups, or cults may experience similar emotional and psychological challenges. We are also aware that a uniform approach may not always be appropriate. For instance, involving family members may not be effective if they hold the same beliefs as the individual, potentially reinforcing the existing mindset rather than challenging it. In such cases, alternative support systems or neutral parties may be more appropriate. Despite these contextual differences, the underlying principles of our interventions remain relevant across various groups facing transitions away from deeply held beliefs. Tailoring interventions to the specific needs and circumstances of the individual is essential for their success and ethical integrity.

## Conclusion

In summary, the current study has provided a platform for four former members of conspiracy theory groups to articulate their experiences within and outside the echo-chamber. Employing reflective thematic analysis, the investigation not only illuminates their individual journeys but also offers insights that can inform potential interventions aimed at negating the proliferation of such ideas. Our findings underscore the necessity for a more sophisticated and respectful approach when engaging with individuals associated with conspiracy theories. Rather than attempting to merely ‘install doubt,’ those involved in reforming individuals should focus on ‘revealing doubts.’ Additionally, our study advocates for an expansion of science education to encompass scientific literacy with the incorporation of counterarguments sourced from platforms like YouTube, emphasizing the importance of not only scientific facts but also the comprehension and navigation of alternative perspectives.
